# Placental Abruption and Partial Placental Prolapse During Induction of Labor in a 31-Year-Old Female With Intrahepatic Cholestasis of Pregnancy: A Case Report

**DOI:** 10.7759/cureus.23995

**Published:** 2022-04-09

**Authors:** Maurice H Dick, Monique Brotman

**Affiliations:** 1 Medicine, Saint James School of Medicine, Arnos Vale, VCT; 2 Obstetrics/Gynecology, West Suburban Medical Center, Chicago, USA

**Keywords:** cesarean, placental prolapse, placental abruption, intrahepatic cholestasis of pregnancy, induction of labor

## Abstract

Placental abruption during induction of labor in females with intrahepatic cholestasis of pregnancy is not exceptionally common and there are no documented reports of placental prolapse following abruption in the literature. The aim of this study is to discuss the possibility of placental abruption and partial prolapse of a low-lying placenta during a prolonged induction of labor in a female with recurrent intrahepatic cholestasis of pregnancy following a cholecystectomy. We describe a 31-year-old G4P3003 female with recurrent intrahepatic cholestasis of pregnancy, with no family history of the condition and surgical history of cholecystectomy, whose induction of labor at 37+3/7 gestational weeks for intrahepatic cholestasis of pregnancy was complicated by placental abruption and partial prolapse of the low-lying placenta. Emergency cesarean section was required for the delivery of her healthy baby. Postpartum was complicated by severe postpartum hemorrhage, post-hemorrhagic anemia, hypotension, blood transfusion reaction, endometritis, and pneumonia. The pathophysiology of intrahepatic cholestasis of pregnancy is not fully understood. Intrahepatic cholestasis of pregnancy increases maternal morbidity, may reoccur in subsequent pregnancies, and is associated with adverse perinatal outcomes. Timely intervention at 37-38 gestational weeks can reduce adverse fetal and maternal outcomes. This case report supports the possibility of 1) a correlation between cholecystectomy and the continued recurrence of intrahepatic cholestasis of pregnancy, 2) placental abruption, and 3) partial prolapse of a low-lying placenta, related to the induction of labor in females with intrahepatic cholestasis of pregnancy. Thus, encouraging further studies to facilitate a greater level of understanding.

## Introduction

Cholestasis is the impairment in bile formation or flows that may be manifested through jaundice, fatigue, and pruritus [1]. Early biochemical evidence of cholestasis consists of increases in serum alkaline phosphatase (ALP), gamma-glutamyl transpeptidase (GGT), and the onset of conjugated hyperbilirubinemia [1]. Cholestatic conditions may be intrahepatic or extrahepatic. Cholestasis is considered chronic if it persists for more than three to six months. The absorption of fat-soluble vitamins is impaired by cholestasis predisposing an individual to metabolic bone disease [1]. Intrahepatic cholestasis of pregnancy and induction of labor complications will be discussed in this report.

Intrahepatic cholestasis of pregnancy is the most common hepatic disorder related to pregnancy in women, occurring in about 1% of pregnancies, and is associated with increased risks of premature deliveries [2]. No long-term effects on the offspring were reported despite the high rates of prematurity but females may have an increased risk of later hepatobiliary cancer and immune-mediated and cardiovascular diseases [2, 3]. To note, the incidence of ICP in liver transplant recipients at the National Transplantation Pregnancy Registry (NTPR) is seven times higher than in the general population [2]. However, recipients reported that intrahepatic cholestasis of pregnancy (ICP) symptoms resolved postpartum, and none indicated continuing transplant problems related to cholestasis of pregnancy (COP).

Intrahepatic cholestasis of pregnancy is a liver condition in the late second and early third trimester. The condition is also known as obstetric cholestasis (OC) or cholestasis of pregnancy COP and is characterized by pruritus of palms and soles (which progresses to generalized intolerable itching) with increased serum bile acids and other liver function tests [4]. ICP is associated with increased risks of stillbirths, respiratory distress syndrome, meconium passage, and fetal asphyxiation [4]. ICP was associated with delivery before 37 gestational weeks, generalized pruritus of the palms and soles, postpartum hemorrhage, meconium-stained liquor, placental abruption, preterm labor, bleeding manifestation, fetal distress, and cord prolapse [5]. Postpartum hemorrhage and placental abruption displayed the most significant increases in those with ICP. The causes of these conditions are considered to be associated with the abnormal placental vascular response to elevated maternal serum bile acids [4]. Bile acids are also considered to influence the onset of preterm labor through the induction of myometrial contractions [6].

Elevated levels of serum bile acids are the most sensitive and specific diagnostic indicator of ICP [6]. A transplacental gradient typically facilitates the clearance of bile acids from the fetus. However, in ICP, maternal bile acids cross the placenta, and accumulate in the fetus and amniotic fluid, impairing the normal transplacental gradient of bile acids [1]. The risks correlate with increasing maternal serum bile levels, particularly when they exceed 40 μmol/L. The clinical and laboratory abnormalities caused by cholestasis of pregnancy generally resolve quickly following delivery but may recur with subsequent pregnancies [1].

## Case presentation

A 31-year-old G4P3003 Caucasian Hispanic female with a penicillin allergy and medical-surgical history significant for cholecystectomy (January 2018), and ICP in her first (2017) and third (2020) pregnancy, presented to her midwife with complaints of itching at 32 weeks gestation. On June 1st, 2021, lab results indicated her total bile acids were elevated at 14.6 μmol/L (normal <11-μmol/L). She was diagnosed with ICP and started on ursodiol 300mg orally two times daily. Prior to this diagnosis, her pregnancy was uncomplicated. She initiated routine prenatal care with her midwife in the first trimester. Her earliest obstetric ultrasound on January 6th, 2021, showed an anterior appearing placenta, a single live intrauterine pregnancy with a size corresponding to 11+3/7 gestational weeks, and, an estimated date of delivery of July 25th, 2021. A follow-up ultrasound on March 2nd, 2021, confirmed the anterior positioned placenta, noted no previa and, a fetus at 19+2/7 weeks gestation.

Her first presentation to the clinic was on June 7th, 2021, to discuss her diagnosis of ICP and a birth plan moving forward. The patient reported no family history of the condition and induction of labor (IOL) in her first and third pregnancies were uncomplicated and resulted in vaginal births of healthy babies at 37 gestational weeks and 37+1/7 gestational weeks, respectively. ICP did not complicate her second pregnancy, nor did she require IOL, resulting in the spontaneous vaginal delivery of a healthy baby at 39+5/7 gestational weeks (November 2018). This present pregnancy, shared decision was made to continue ursodiol 300mg orally two times daily for the duration of the pregnancy, and the IOL at 37 gestational weeks. Her visit also addressed new complaints of hematuria, menstrual-like cramps, and urinary frequency and urgency. A clean-catch urine culture was obtained and found to be abnormal for Escherichia coli. She was successfully treated with cephalexin 500 mg orally every six hours for seven days and nitrofurantoin microcrystal 100mg orally two times daily for seven days.

**Figure 1:**
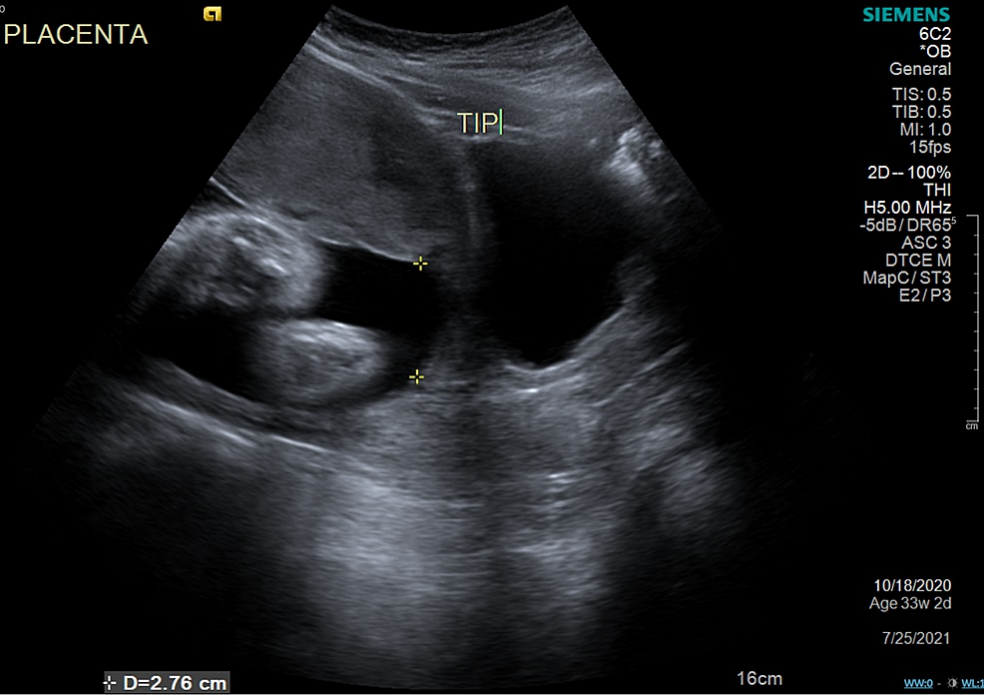
Transabdominal ultrasound measuring the placenta edge to be 2.76 cm from the internal cervical os.

The patient completed an obstetric ultrasound on June 8th, 2021, where the placental edge was measured to be 2.76 cm from the internal cervical os (Figure 1), and the fetal measurements noted a 33+2/7 gestational weeks intrauterine pregnancy. Repeat total bile acid test on June 23rd, 2021, showed a reduction to 2.8 μmol/L.

Late into the night of July 7th, 2021, the patient presented to labor and delivery for her scheduled induction of labor at 37+3/7 weeks gestation. An assessment revealed: an estimated fetal weight of 6.5-7 lbs by Leopold’s, irregular uterine contractions on tocodynamometer (toco), Category I Fetal Heart Tracing (FHT), and cephalic presentation confirmed via sonography. A sterile vaginal exam revealed a thick, soft, high, and posterior cervix. IOL was started with the placement of a Cook balloon catheter and intravenous Pitocin at 2 mU/min for the duration of balloon placement.

On July 8th, 2021, no significant changes were noted and Cytotec was given for cervical ripening.

On July 9th, 2021, at 0615 hours, the membrane spontaneously ruptured. At 1400 hours, sterile vaginal examination revealed 0 stations, and a cervix 9 cm dilated and 100% effaced. At 1432 hours, the patient reported a big gush of fluid. Upon inspection, significant frank red blood was noticed on the underpads, and on sterile vaginal examination, the placenta was palpated within the vaginal vault with no fetal presenting part. The patient was taken immediately to the operating room for an emergency C-section. Upon hysterotomy, portions of the placenta extruded from the incision and were not attached to the uterus. At 1456 hours, a live infant weighing 6.7 lbs and Apgar’s 9/9 was delivered with vacuum assist without issue. The patient suffered severe postpartum hemorrhage with an estimated blood loss of 2,360 cc. During the procedure, she was given 2,000 cc of Lactated Ringer’s, and her urine output was 675 cc. Postoperatively, the patient was given one unit of packed red blood cells (pRBC) and one unit of fresh frozen plasma (FFP). Her postpartum course was also complicated by post-hemorrhagic anemia, hypotension, blood transfusion reaction, endometritis, and pneumonia.

**Figure 2:**
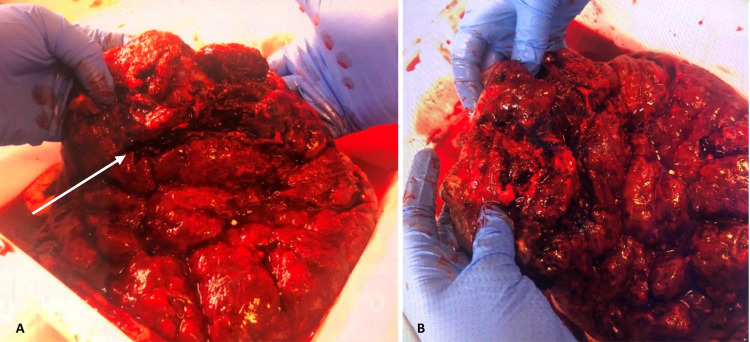
A) Arrow points to a fissure created on the maternal surface when 1/4 to 1/3 prolapsed into the vaginal vault, while B) shows the disrupted maternal surface with adherent clots in the prolapsed portion.

Concerned for abruption as the cause of the prolapse, the placenta was examined after the operation, and it was noted 1/4 to 1/3 of the placenta (with adherent clots) separated from the rest of the placenta (Figure 2). The pathology report cited the maternal surface to be disrupted, and when reconstituted, complete cotyledons with slight possible gray-tan calcifications were identified. On sectioning, slightly firm areas were noticeable ranging from 0.4 to 0.6 cm. The remaining placental disc revealed the usual red-tan spongy maternal surface with no additional placental parenchymal lesions identified.

## Discussion

The etiology and pathogenesis of ICP are determined by several factors, including genetic, hormonal, and exogenous [7]. A high incidence of the recurrence of ICP is more common among women with a family history of the condition (9.2%) than those with none (4.2%) [7]. Genetically predisposed women possess large amounts of sulfated progesterone metabolites in pregnancy which may result in the saturation of the hepatic transport system utilized for the biliary excretion of these compounds. Elevated levels of estrogens and progesterone are associated with cholestasis with an increase in the bile acid levels. Estrogen and progesterone can also regulate genes resulting in reduced bile acid transport in susceptible women [7].

Genetic defects in transporters that control the export of bile acids from hepatocytes to biliary canaliculi are also associated with ICP. Mutations in the genes encoding for bile acids and phospholipid transporters (ABCB4/MDR3, ATP8B1/ F1C1, ABCB11/BSEP, ABCC2) are mainly associated with ICP [7]. ABCB4/MDR3 help to protect cells from bile salts which mainly cause hepatic cell injury and cholestasis. Mild dysfunction of these transporters may not cause any problem in the absence of pregnancy; however, it could result in cholestasis when their capacity to secrete is exceeded by the excessive levels of hormones produced during pregnancy [7]. Environmental factors such as low vitamin D (caused by inadequate exposure to sunlight) and dietary factors such as minimized selenium levels are also associated with ICP. Selenium is utilized in various enzymatic activities in the liver during hydroxylation of cholesterol, and its low levels may hinder the normal bile acid metabolism [7].

The diagnosis of ICP is associated with elevated levels of bile acids and the exclusion of other causes of liver diseases [7]. Differential diagnosis of ICP includes viral hepatitis, biliary obstruction, primary biliary cirrhosis, primary sclerosing cholangitis, and liver diseases specific to pregnancy (preeclampsia, hemolysis, elevated liver enzymes, low platelets syndrome, and acute fatty liver of pregnancy). Some dermatological conditions may present with pruritis in pregnancy, but the liver function tests are normal, and bile acids levels are not raised. Bile acids should be less than 10 μmol/L during normal pregnancy [7].

Mono treatment with ursodeoxycholic acid (UDCA), also known as ursodiol, had no significant effect on the prevalence of stillbirth in women with ICP nor does it reduce adverse perinatal outcomes [8]. However, some studies showed UDCA was associated with a reduction in stillbirth in combination with preterm birth, providing evidence for the clinical benefit of the treatment [8]. UDCA is essential in improving biliary flow, enhancing the protective bicarbonate environment on the surface of cholangiocytes, and cushions the liver from bile acid-induced apoptosis [8]. The treatment can reduce the elevation of serum bile acid concentration in the fetus (by regulating placental bile acid export) and has anti-inflammatory actions [8].

Clinical treatment of ICP is aimed at improving patient symptoms and fetal prognosis [9]. Thus, UDCA is the first-line drug for the treatment of ICP and is essential in improving the pregnancy outcomes of patients with ICP [9, 10]. The drug is characterized by safety, effectiveness, high compliance, and convenience and is widely used in clinical practice [9]. However, the drug regulatory authorities have not yet approved UDCA as a pregnancy-safe drug, which accounts for the reluctance of obstetricians to use it on pregnant patients [10]. Some obstetricians utilize other medications such as rifampin, cholestyramine, and S-adenosyl-L-methionine [4]. Rifampin is essential in increasing bile acid detoxification and excretion and can be used as an adjunct to UDCA [4]. Cholestyramine, an anion exchange resin, is crucial in decreasing the ileal absorption of bile salts, thus increasing their fecal excretion. S-adenosyl-L-methionine is prescribed using a twice-daily intravenous regimen making it less utilized in the management of ICP. Pruritis caused by cholestasis of pregnancy can be managed using antihistamines such as chlorpheniramine. Antihistamines do not affect serum bile acids but may be essential in reducing the sensation of pruritus in some women [4].

If treatment with UDCA fails, premature delivery should be considered [11]. The risk of intrauterine fetal hypoxia and mortality is higher after the 38th gestational week (even in cases of moderate cholestasis), thus IOL at 37 gestational weeks is recommended [11]. Some studies suggest delivery may be initiated at an earlier gestational period, most feasible 35 to 37 gestational weeks, in patients with bile acid levels of 100 mmol/L [7]. IOL is essential when the risk of continuing with the pregnancy outweighs that of labor induction and delivery for the parent or the fetus [12]. Corticosteroids such as Misoprostol (Cytotec) and Oxytocin (Pitocin) should be administered before the 34th week of gestation to reduce fetal mortality, intraventricular bleeding, and respiratory insufficiency [7]. IOL is performed to minimize the risk of preterm birth, neonatal respiratory distress syndrome, meconium-stained amniotic fluid, and stillbirth, among other complications that may arise from the condition [13]. IOL among women with cholestasis of pregnancy is a common practice and delivery at 36 gestational weeks is essential in the reduction of perinatal mortality risks as compared to expectant management [14].

In women who do not have ICP, IOL is conducted in cases where the delivery is overdue, or the pregnancy may pose health risks for the mother or the fetus [15]. IOL in women with ICP may also contribute to an increased risk of emergency C-sections, newborn complications, and low fetal birth weight as delivery is induced early. Placental cord prolapse can occur during IOL in women with ICP. Placental cord prolapse occurs when the umbilical cord exits the cervical opening before the fetal presenting part [16]. Intrapartum interventions such as cervical ripening, labor induction, amniotomy, and amnioinfusion are associated with cord prolapse [16]. Compression of the cord by the descending fetus during delivery leads to fetal hypoxia and bradycardia, resulting in fetal mortality or morbidity [16]. A low-lying placenta within 2 cm of the cervical os is not a contraindication for a trial of labor and the morbidity in these women did not increase [17].

Placental abruption can also occur during labor induction in women with ICP and is the leading cause of maternal morbidity and perinatal mortality [18]. Placental abruption involves the early separation of the placenta from the uterine lining before completing the second stage of labor [18]. Depending on the distance of the placenta from the cervical os, further complications can arise if a low-lying placenta enters the vaginal vault after abruption. This complication can be termed, placental prolapse. Placental abruption may be associated with renal failure, hysterectomy, the risk of hemorrhage and the need for blood transfusions, postpartum pituitary gland necrosis, Sheehan syndrome, and disseminated intravascular coagulopathy [18]. IOL in women with and without ICP may cause high rates of postpartum hemorrhage, bladder injury, fetal injury, sepsis, scar disunion, intensive care unit transfer, infection, and phlebitis [19]. The risk of postpartum hemorrhage is associated with the use of prostaglandin following a transcervical catheter [19]. In both women with ICP and those without, IOL may be associated with various complications. However, in women with ICP, timely intervention at 37-38 gestational weeks will reduce adverse outcomes for the mother and the baby [20].

## Conclusions

Cholestasis is the impairment in bile formation or flows that may be manifested through jaundice, fatigue, and pruritus. ICP is a liver condition in the late second and early third trimesters. It occurs in about 1% of pregnancies and is associated with increased risks of premature deliveries. ICP may reoccur in subsequent pregnancies and with a higher incidence among women with a family history of the condition. The diagnosis of ICP is associated with elevated levels of bile acids and the exclusion of other causes of liver diseases. Clinical treatment of ICP is aimed at improving patient symptoms and fetal prognosis. A low-lying placenta is not a contraindication for a trial of labor. UDCA, Misoprostol, and Oxytocin may be used to treat ICP. IOL may cause several complications in women with ICP and those without the condition, however, timely intervention at 37-38 gestational weeks will reduce adverse outcomes for the mother and the baby. This case report supports the possibility of 1) continued ICP after cholecystectomy, 2) placental abruption, an obstetric emergency, and subsequent 3) placental prolapse (depending on the distance of the placenta’s edge to the cervical os), related to the IOL in females with ICP. Thus, encouraging further studies to facilitate a greater level of understanding.

## References

[1] Hilscher MB, Kamath PS, Eaton JE (2020). Cholestatic liver diseases: a primer for generalists and subspecialists. Mayo Clin Proc.

[2] Coscia L, Moritz MJ, Armenti DP, Constantinescu S (2016). Cholestasis of pregnancy in liver transplant recipients. Am J Obstet Gynecol.

[3] Wikström Shemer EA, Stephansson O, Thuresson M, Thorsell M, Ludvigsson JF, Marschall HU (2015). Intrahepatic cholestasis of pregnancy and cancer, immune-mediated and cardiovascular diseases: a population-based cohort study. J Hepatol.

[4] Pillarisetty LS, Sharma A (2019). Pregnancy intrahepatic cholestasis. StatPearls [Internet].

[5] Dodampahala SH, Pieris H, Chandrasena LG (2016). Presence of obstetrics cholestasis in mothers presenting with pruritus in pregnancy: a low resource south Asian setting. ARSci.

[6] Chivers S, Williamson C (2018). Intrahepatic cholestasis of pregnancy. Obstet Gynaecol Reprod Med.

[7] Kumar S, Puri P, Gujral K (2018). Intrahepatic cholestasis of pregnancy. Current Medicine Research.

[8] Ovadia C, Sajous J, Seed PT (2021). Ursodeoxycholic acid in intrahepatic cholestasis of pregnancy: a systematic review and individual participant data meta-analysis. Lancet Gastroenterol Hepatol.

[9] Roy A, Premkumar M, Mishra S (2021). Role of ursodeoxycholic acid on maternal serum bile acids and perinatal outcomes in intrahepatic cholestasis of pregnancy. Eur J Gastroenterol Hepatol.

[10] Parízek A, Simják P, Cerný A (2016). Efficacy and safety of ursodeoxycholic acid in patients with intrahepatic cholestasis of pregnancy. Ann Hepatol.

[11] Piechota J, Jelski W (2020). Intrahepatic cholestasis in pregnancy: review of the literature. J Clin Med.

[12] Coates D, Makris A, Catling C (2020). A systematic scoping review of clinical indications for induction of labour. PLoS One.

[13] Arthuis C, Diguisto C, Lorphelin H, Dochez V, Simon E, Perrotin F, Winer N (2020). Perinatal outcomes of intrahepatic cholestasis during pregnancy: an 8-year case-control study. PLoS One.

[14] Friberg AK, Zingmark V, Lyndrup J (2016). Early induction of labor in high-risk intrahepatic cholestasis of pregnancy: what are the costs?. Arch Gynecol Obstet.

[15] Cherney Cherney, k. (2019 (2021). Everything you need to know on labor induction. https://www.healthline.com/health/pregnancy/inducing-labor#side-effects.

[16] Boushra M, Stone A, Rathbun KM (2020). Umbilical cord prolapse. StatPearls [Internet].

[17] Jansen C, de Mooij YM, Blomaard CM (2019). Vaginal delivery in women with a low-lying placenta: a systematic review and meta-analysis. BJOG.

[18] Schmidt P, Skelly CL, Raines DA (2020). Placental abruption. StatPearls [Internet].

[19] Delporte V, Grabarz A, Ramdane N, Bodart S, Debarge V, Subtil D, Garabedian C (2019). Cesarean during labor: is induction a risk factor for complications?. J Gynecol Obstet Hum Reprod.

[20] Hassan N, Khurshid R, Muzamil M, Parveen S (2020). Cholestasis of pregnancy: the effects on maternal and fetal outcome. Int J Reprod Contracept Obstet Gynecol.

